# Undeserved authorship in surgical research: an underestimated bias with potential side effects on academic careers

**DOI:** 10.1007/s13304-023-01581-w

**Published:** 2023-07-13

**Authors:** Arcangelo Picciariello, Agnese Dezi, Donato F. Altomare

**Affiliations:** Surgical Unit M. Rubino, Department of Precision and Regenerative Medicine and Ionian Area (DiMePRe-J), University Aldo Moro of Bari, Azienda Ospedaliero Universitaria Policlinico Bari, Piazza G. Cesare, 11, 70124 Bari, Italy

**Keywords:** Courtesy authorship, Surgical research, Gift authorship, Academic surgery

## Abstract

The incidence of courtesy authorship in research over time has probably increased due to the enormous pressure to publish to increase the bibliometric indexes necessary to achieve an academic role. The aim of this survey was to quantify and characterize this research malpractice among a very selected group of surgeons from different surgical specialties belonging to the European Association of Surgery (ESA). E-mail addresses for the invitation to take part to the survey were collected by the Twenty-eighth Annual Meeting final program. Five-item were designed and developed by the authors using an online platform. Eighty-six members from 21 countries completed the survey (female/male ratio: 0.09). In the last 10 years, almost half of the responders (41, 47.7%, 37 academics) have included colleagues for courtesy authorship. The most common reason of courtesy authorships was to support the academic career of another researcher (62.5%). Other reasons were fear of retaliation (12.5%), reciprocal authorship (12.5%) or support for a partner (10%). This survey showed that undeserved authorship is sadly confirmed to be a common research misconduct across any countries and medical specialties, even among a very selected group of surgeons with international reputation irrespective of the academic position.

## Introduction

Writing a research paper is not too different from other products of human ingenuity, such as a piece of music or a movie, but its intellectual property is not adequately protected, even by the authors of a publication, who gladly share the paternity of their work with other colleagues who did not play an active or adequate role to its creation. This common practice, known as courtesy authorship, is often considered “victimless”; nevertheless, it can deeply damage the authors themselves or other colleagues when competing for an academic position or a grant for research [1].

There are several shades of this phenomenon known as courtesy authorship. *Guest authorship* occurs when senior authors are included because of their respect or influence in the hope that this will increase the likelihood of publication and/or impact of the paper once published [2]; *honorary authorship*, instead, refers to those who are named authors merely, because they hold senior positions within the service or facility where the research occurred, and may have helped secure funding [3]. Finally, the most common one is the *gift authorship* which could be motivated by several reasons, including economic interests, mutual agreements between two researchers (who do not work together) to add each other to their respective lists of authors, or support your colleagues, partners or family members to favor their academic career, etc. [4].

On the contrary, *ghost authorship* is another undetectable malpractice, where someone who contributed actively in the research is not disclosed in the author’s byline [5].

The burden of this malpractice in research is probably underestimated, and therefore, we conducted an anonymous survey among the members of the ESA, the European Association of Surgery, one of the most reputative surgical association in Europe. Members of this society are considered the elite of surgeons from different specialties in Europe, since their admission is decided by the member of the ESA on the base of the relevance of their surgical research and on their impact on clinical practice. Therefore, one should expect those ESA members to be almost free from this practice.

The aim of this survey is to quantify and characterize this research malpractice among a very selected group of European surgeons from different surgical specialties.

## Methods

Well-known expert surgeons and researchers belonging to the ESA (European Surgical Association) were invited to complete an online anonymous survey in the period between September and November 2022. Data regarding country, age, gender and job position (academic or not) were collected.

E-mail addresses for the invitation to take part to the survey were collected by the Twenty-eighth Annual Meeting final program.

Five-items were designed and developed by the authors using an online platform (“Google Form”) (Table 1).

**Table 1:** Structured questionnaire administered

Country	Select your country
Age	Type your age
Gender	Male
	Female
Are you aware of the International Committee of Medical Journal Editors (ICMJE) authorship criteria?	Yes
	No
In the last 10 years have you ever received a **guest authorship**? DEFINITION: Guest authorship refers to senior authors who are included because of their respect or influence in the hope that this will increase the likelihood of publication and/or impact of the paper once published	Yes
	No
In the last 10 years have you ever received an **honorary authorship**? DEFINITION: Honorary authorship refers to those who are named as authors merely because they hold senior positions within the service or facility where the research occurred, and may have helped secure funding	Yes
	No
In the last 10 years have you ever received a **gift authorship**? DEFINITION: GIFT AUTHORSHIP would include mutual agreements between two researchers (who do not work together) to add each other to their respective lists of authors, in order to increase their publication numbers, or where a researcher may feel obliged to provide authorship to current or former colleagues to repay help or mentorship received	Yes
	No
In the last 10 years have you ever included colleagues for courtesy authorship in your papers?	Yes
	No
If you have included colleagues for **courtesy authorship** please click the reason	Family members
	Partners
	Money
	To support academic career
	To please the head of the Dept
	To obtain a reciprocal authorship
	Fear of retaliation
	Other reasons (please specify)

The survey aimed to capture the current status of different types of gift authorship in surgery.

Surgeons who did not respond to the first e-mail invitation were contacted by a second e-mail.

Understanding of ICMJE authorship criteria [6] was checked by the first item.

In items 2, 3 and 4, the definition of guest, honorary and gift authorship was provided since they are subtypes of courtesy authorship.

The last item evaluated the percentage of courtesy authorships in the last 10 years.

When the surgeons reported having added a courtesy author in the past, they were directed to a further item investigating the reasons.

Initial exploratory analysis was performed using standard descriptive statistics including mean or medians for continuous data and categories with raw numbers and percentages for categorical data.

## Results

Out of 221 emails sent, 17 were returned because of delivery system errors. Eighty-six members from 21 countries completed the survey (42.2%). Mean age was 60.4 (range 43–83) and female/male ratio was 0.09 (79 males and 7 females).

Most responders (82, 95.3%) were academics working as associate (11) or full Professors (71).

Only 75 members (87.2%, 71 academics) declared to be aware of the International Committee of Medical Journal Editors (ICMJE) authorship criteria.

Regarding “guest authorship”, 21 participants (24.4%, 20 academics) confirmed having received at least one guest authorship in the last 10 years.

Twenty surgeons (23.2%, 19 academics) were named authors merely, because they hold senior positions within the service or facility, where the research occurred.

Some of the responders (9, 10.4%, 8 academics) admitted to have had mutual agreements with other researchers (who do not work together) to add each other to their respective lists of authors to increase their publication number.

In the last 10 years, almost half of the responders (41, 47.7%, 37 academics) have included colleagues for courtesy authorship.

**Fig. 1:**
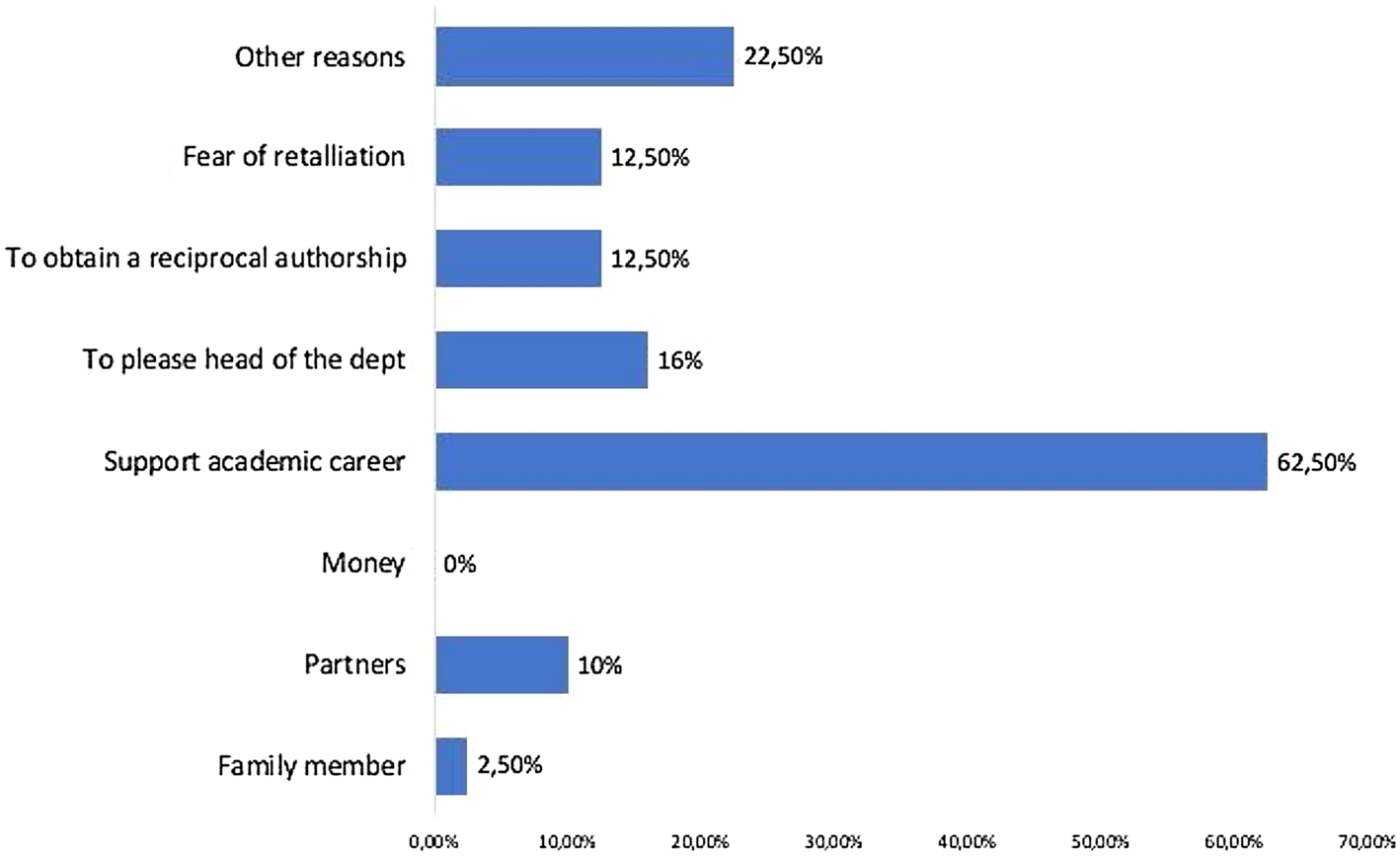
Percentage of reasons for the gift authorship

The most common reason of courtesy authorships was to support the academic career of another researcher (62.5%). Forty percent of the participants recurred to courtesy authorship to please the head of the department. Other reasons were fear of retaliation (12.5%), reciprocal authorship (12. 5%) or support for a partner (10%). Surprisingly, only three surgeons indicated the participation to multicenter studies as the reason for a courtesy authorship. No one mentioned money among the reasons for the gift authorship (Fig. 1).

## Discussion

Researchers with a large number of publications are usually considered to have more authority and expertise, and, therefore, are favored in academic competition. Therefore, this “Publish or Perish” culture has triggered a dissolute rush to publish the largest number of papers in the shorter time possible favoring the practice of unethical medical writing and manipulation of the authorship criteria.

Our anonymous survey shows that undeserved authorship is sadly confirmed to be a common research misconduct across any countries and medical specialties, even among a very selected group of surgeons with international reputation irrespective of the gender and academic position, despite the consideration that some degree of reluctance to admit an improper authorship could be expected even if the survey was anonymous.

The most common type of gift authorship among this selected group of surgeons is the guest authorship motivated by holding a senior position in the team or to achieve reciprocal citations.

On the other hand, in agreement with other authors [7], the support of other researchers to speed their academic career was the most common justification for this type of authorship.

Interestingly, economic interest was never declared among this group of scientists and the wish to support partners (*son and lovers’ rule*) was very unfrequently reported, despite the common sensation that it represents a common practice.

A recent paper investigates courtesy authorship practice among first and senior authors of publication in eight surgical journals during a timeframe of 1 year, reporting that, as expected, this common practice was more frequent in academic setting (75%), where publication is closely related to progress in academic career, and that it occurs even more than five times during the same year in a considerable percentage of cases [7].

The incidence of courtesy authorship in research over time has probably increased due to the enormous pressure to publish to increase the bibliometric indexes necessary to achieve the role of professor [8], and seems to be more frequent in papers published in low impact factor journals than in high impact factor journals.

An interesting survey by Slone states that only the first two authors account for all the criteria for authorship, while all the others are listed according to their decreasing relevance [9]. This could mean that the final author, who is commonly accredited to be the most authoritative one, is often an honorary authorship.

On the other hand, it must be recognized that many modern studies involve authors from several different specialties who effectively contributed to the paper by means of their knowledges and skillness thus justifying their role as authors.

The phenomenon of courtesy authorship has exploded during the COVID19 pandemic due to the push to publish as soon as possible vital information for understanding and managing the pandemic [10]. In fact, a multitude of multicenter studies involving hundreds or even thousands of “authors” have been published in that period, analyzing big data recruited in a very short period of time [11]. Of course, the true authors of these papers are a minority of the long list of names who have contributed by sharing their clinical data, but without fulfilling the requisites for authorship claimed in almost all the journals [12]. Even the prestigious Cochrane database reviews are not immune from this unacceptable practice [13].

Attempts to limit this fraudulent practice have been proposed by several studies, including the limitation to the number of authors to be included and detailed description of the role of each author and the potential conflict of interests in the study, but, so far, without significant results.

In conclusion, this survey confirms the relevance of the bad practice of courtesy authorship, which is very common even among a very selected group of surgical scientists, representing a plague in medical research.

## Data Availability

Data are available on request from the correspondimg author A.P.

## References

[1] Varghese J, Jacob M (2022). Gift authorship: look the gift horse in the mouth. Indian J Med Ethics.

[2] Morreim EH, Winer JC (2023). Guest authorship as research misconduct: definitions and possible solutions. BMJ Evid Based Med.

[3] Quaia E, Crimi F (2021). Honorary authorship: is there any chance to stop it? Analysis of the literature and a personal opinion. Tomography.

[4] Panta P (2022). Gift authorship and ways to subdue it. Br J Oral Maxillofac Surg.

[5] Pruschak G, Hopp C (2022). And the credit goes to …—Ghost and honorary authorship among social scientists. PLoS ONE.

[6] (2010) Uniform requirements for manuscripts submitted to biomedical journals: writing and editing for biomedical publication. J Pharmacol Pharmacother 1(1):42–58 PMC3142758 21808590

[7] Condron ME (2021). Courtesy authorship practices among first and senior authors: evaluation of motivations, gender bias, and inequities. Ann Surg.

[8] McClellan JM (2019). Courtesy authorship in academic surgery publications. JAMA Surg.

[9] Slone RM (1996). Coauthors’ contributions to major papers published in the AJR: frequency of undeserved coauthorship. AJR Am J Roentgenol.

[10] Picciariello A, Gagliardi G, Altomare DF (2020). It’s COVID o’clock. Br J Surg.

[11] Picciariello A, Altomare DF (2021). Big data papers and COVID-19: a “false friend” for academic surgeons. Tech Coloproctol.

[12] Altomare DF (2019). Defining authorship in the era of big data collection and its consequences on the academic career. Int J Colorectal Dis.

[13] Gulen S (2020). More than one-third of Cochrane reviews had gift authors, whereas ghost authorship was rare. J Clin Epidemiol.

